# Hiding in Plain Sight: An Unusual Case of Progressive Dysphagia, Dyspnea and Dysphonia

**DOI:** 10.1007/s00455-020-10183-2

**Published:** 2020-09-01

**Authors:** Stephanie D. Mes, Tjouwke A. van Kalkeren, Rutger J. Jacobs, Inez M. J. H. Coene, Antonius P. M. Langeveld, Heiko Locher

**Affiliations:** 1 Department of Otorhinolaryngology, Leiden University Medical Centre, Albinusdreef 2, 2333 ZA Leiden, The Netherlands; 2 Department of Otorhinolaryngology, Alrijne Hospital, Houtlaan 55, 2334 CK Leiden, The Netherlands; 3 Department of Gastroenterology, Alrijne Hospital, Houtlaan 55, 2334 CK Leiden, The Netherlands

## Clinical Conundrum

**Fig. 1:**
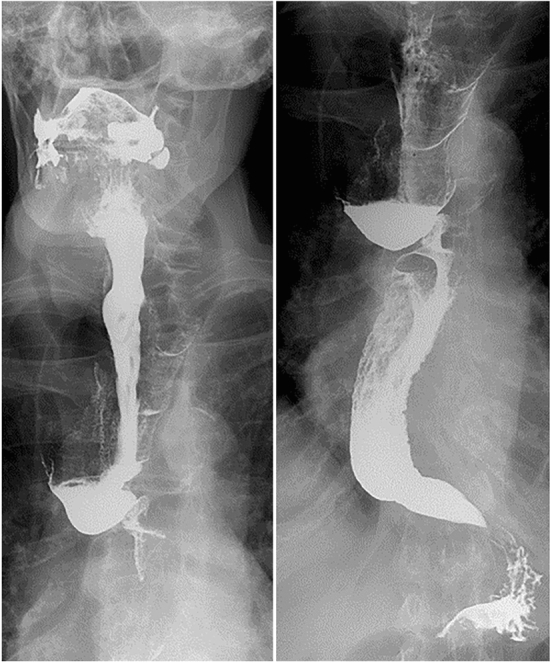
A barium swallow test (anterior–posterior projection) which shows dilatation of the esophagus, a tortuous stenosis at 25 cm, pooling at the lower esophagus and a rat-tail appearance of the esophagogastric junction

**Fig. 2:**
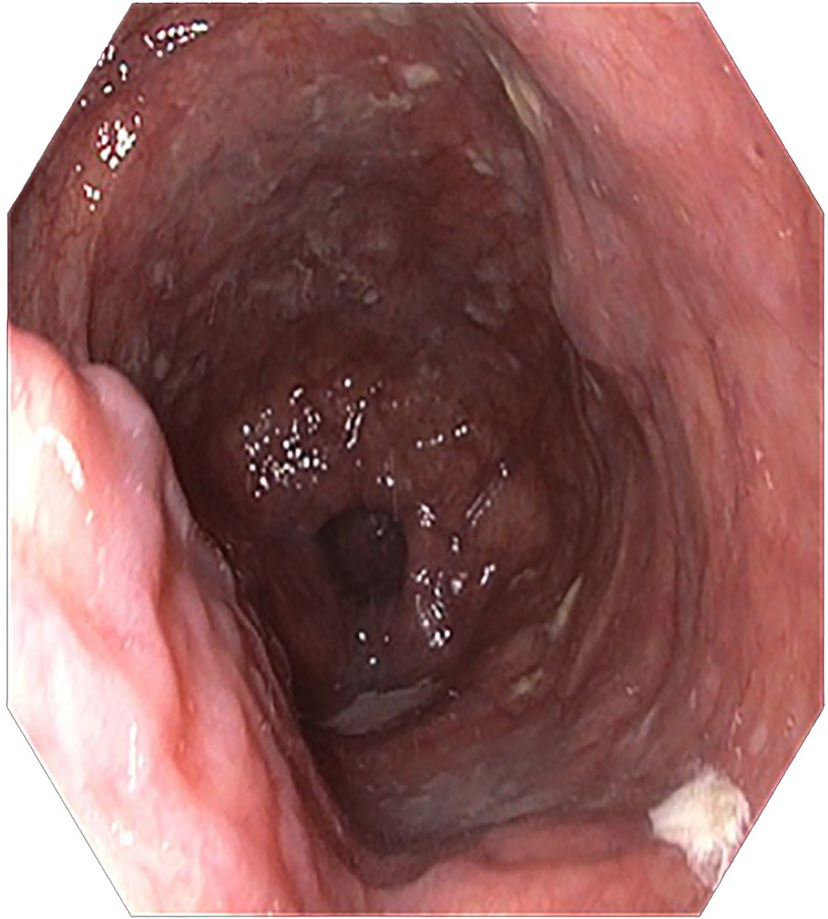
Endoscopic view of the dilated esophagus

An 87-year-old woman presented with progressive solid food dysphagia that had been on-going for over 10 years. Her medical history included sarcoidosis, atrial fibrillation, hypertension, and a right-sided hemicolectomy for cecal adenocarcinoma. During gastroenterological consultation in a secondary setting, a barium swallow test revealed severe dilation of the proximal and distal esophagus, stasis of the bolus at the level of the aortic arch, and a “rat-tail” appearance of the esophagogastric junction (Fig. 1). Subsequent gastroscopy showed atony, esophageal dilation, and a narrowed tortuous segment in the distal esophagus (Fig. 2). Dilation was performed with Savary bougies up to 16 mm; however, this did not improve her swallowing. Although no definitive diagnosis could be made, a long-standing non-specific esophageal motility disorder was suspected. As her symptoms were mild, the patient agreed to conservative management with observation.

**Fig. 3:**
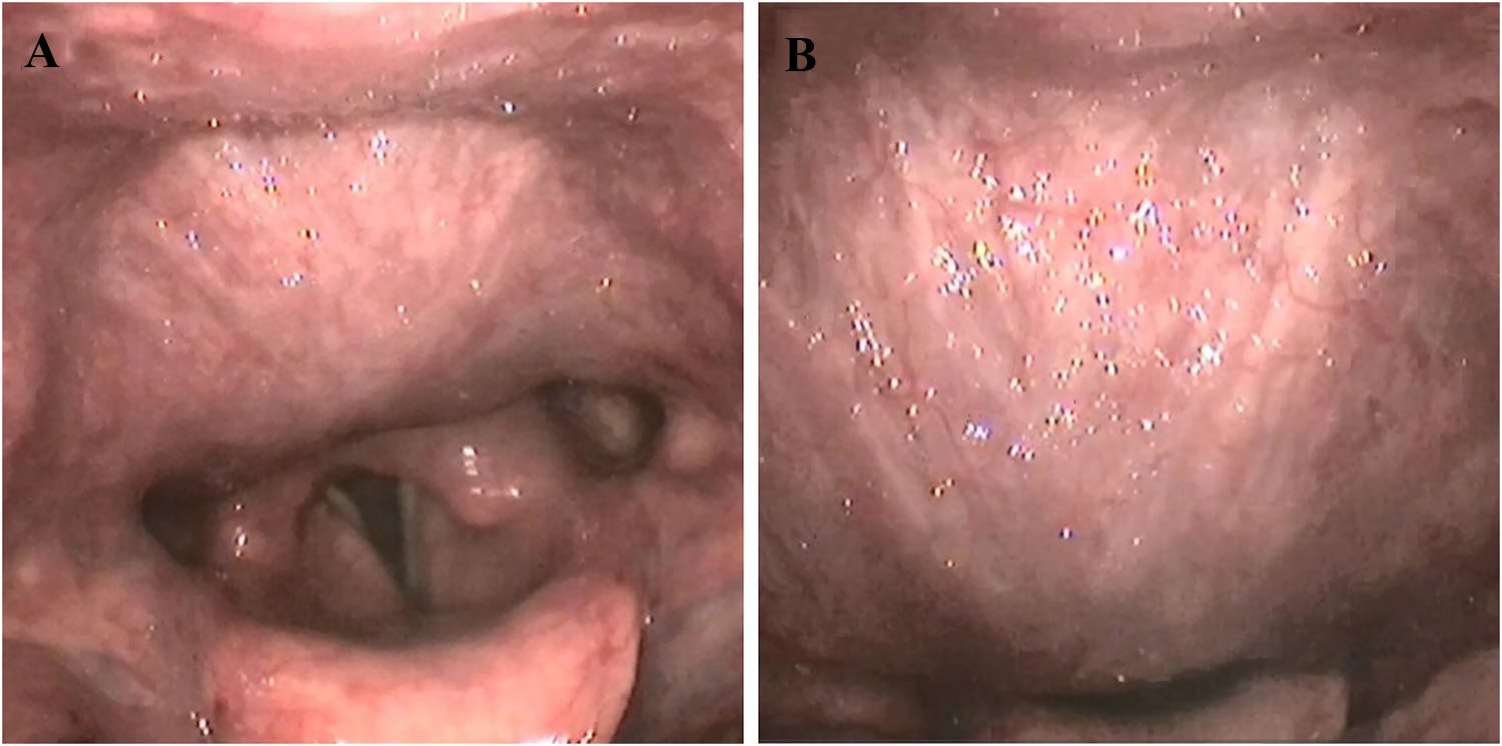
Flexible laryngoscopy shows a ball-shaped submucosal swelling of the posterior laryngopharyngeal wall (**a**). The swelling is progressive when performing Valsalva maneuver, enlarging supra-laryngeal in anterior direction towards the epiglottis (**b**)

**Fig. 4:**
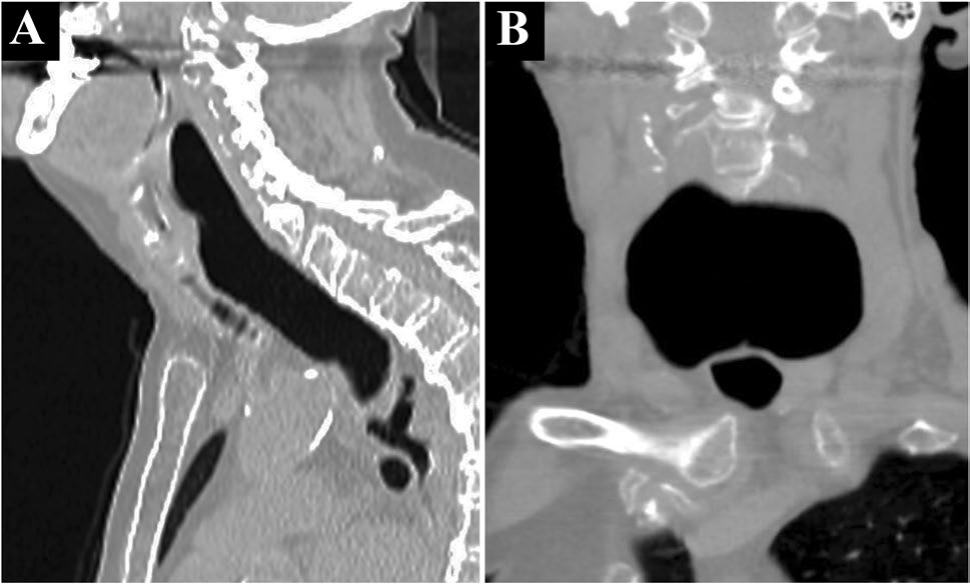
CT scan of the neck, in lateral (**a**) and anterior–posterior (**b**) direction, showing the large dilatation of the esophagus

Two years later, the patient developed additional symptoms of dyspnea and dysphonia. During meals, she became increasingly short of breath. She also developed a dorsal articulation that was progressive during the day. At this time, the patient was referred for tertiary ENT consultation. Fiberoptic laryngoscopy revealed a non-solid bulging of the posterior pharyngeal wall, which was present at rest (Fig. 3a) and expanded over the larynx upon valsalva, swallowing, and phonation (Fig. 3b). CT scanning revealed a severely dilated esophagus (Fig. 4). As the findings did not explain the pharyngeal posterior wall swelling, and since her symptoms had progressed, a second evaluation in a multidisciplinary setting was arranged.

## Teaching and Clinical Points

**Fig. 5:**
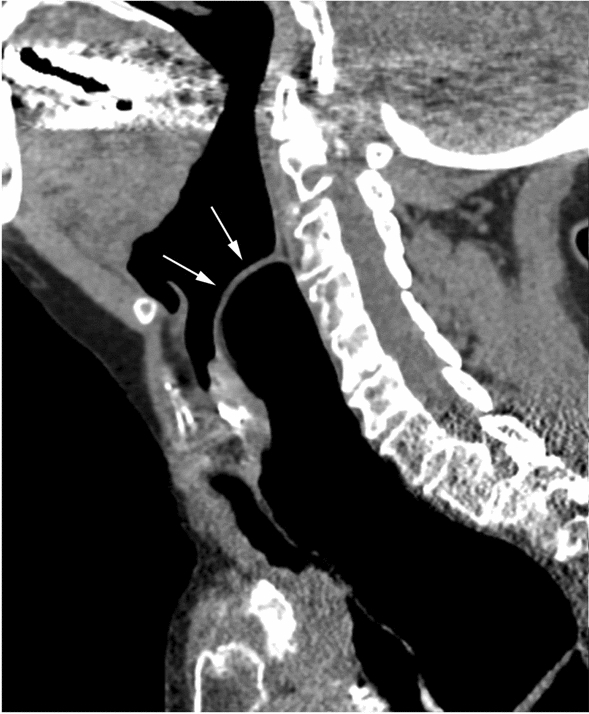
The CT scan of Fig. 4a, here presented after adjusting image contrast settings. The arrows point to the cranial demarcation of the mid-esophageal diverticulum

The multidisciplinary evaluation included a gastroenterologist, radiologist, and otorhinolaryngologist. This group re-evaluated the neck CT scan. Adjustment of the contrast settings revealed a giant mid-esophageal diverticulum, rather than the supposed severely dilated esophagus. The diverticulum expanded in the retropharyngeal space all the way up to the level of the epiglottis tip—measuring 78 mm in the lateral, 38 mm in the antero-posterior, and 140 mm in the cranio-caudal direction (Fig. 5). The posterior pharyngeal wall bulging corresponded to the most cranial part of the air-filled diverticulum. This phenomenon explained her dyspnea and dysphonia during moments of increased intra-thoracic and abdominal pressure (Fig. 3b).

**Fig. 6:**
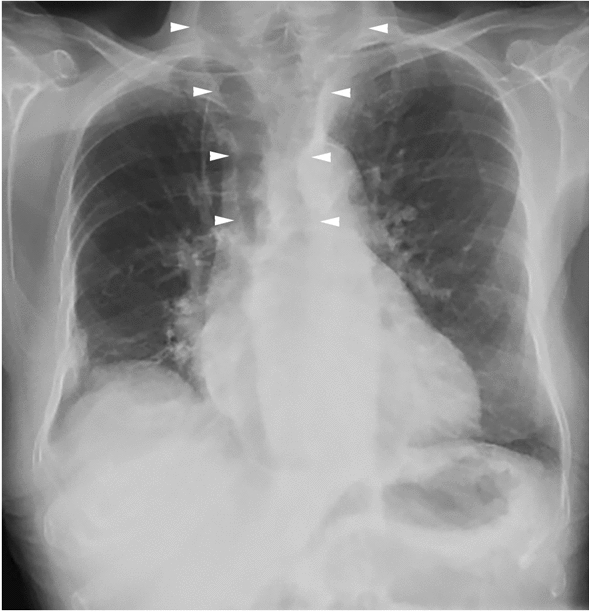
Chest X-ray in anterior–posterior direction, where the air-figure of the diverticulum is demarcated with arrowheads

In hindsight, this diverticulum was readily visible on 15 different chest X-rays that had been taken over the past 10 years for various reasons, and evaluated by different specialists (an example is presented in Fig. 6). Strikingly, with knowledge of the diverticulum’s size, it could also be observed on the original barium swallow test (Fig. 1). This clinical conundrum demonstrates the added value of multidisciplinary evaluations in difficult cases, where the input of various medical specialists is required to find the correct diagnosis.

## Discussion

Mid-esophageal diverticula are generally a few centimeters in size, although a few giant diverticula of up to 8–9 cm have been described in literature. Causes of mid-esophageal diverticula may include traction from mediastinal inflammatory lesions, and pulsion due to esophageal motility disorders. The present case exhibited evidence supporting both of these pathophysiological mechanisms.

Firstly, our patient had sarcoidosis with mediastinal lymph node involvement. The diverticulum could have been formed due to adhesions between the inflamed lymph nodes and the esophagus. Upon esophageal contraction, the adhesions may have pulled on the esophageal wall, eventually leading to formation of a localized diverticulum. This is an example of a true diverticulum, involving all layers of the esophageal wall [1]. Traction diverticula are most commonly seen in cases of histiocytosis or tuberculosis, but have been also described in relation to sarcoidosis [2, 3].

Secondly, pulsion mid-esophageal diverticula tend to be associated with esophageal motor disorders, which are predominantly non-specific [4]. In such cases, increased pressure on the esophageal lumen causes herniation of the mucosa and submucosa through the esophageal musculature. Our present patient exhibited a tortuous stenosis directly below the origin of the giant diverticulum. Therefore, it can be argued that both pathophysiological mechanisms played a role in the formation of our patient’s diverticulum.

When symptomatic, diverticula can be surgically removed using either an open or laparoscopic approach. In this case, due to the diverticulum size, our patient’s age, her wishes and her progressive cardiac failure, it was decided to forgo diverticulum removal surgery after shared decision-making. As a symptomatic treatment for her respiratory symptoms a tracheostomy was offered. However, our patient also preferred not to undergo this treatment (she did not want any procedures done anymore) and stayed therefore somewhat limited in her activity and mobility.

In this clinical conundrum, we presented the case of a giant mid-esophageal diverticulum that caused dysphagia, dyspnea, and dysphonia, and was initially overlooked due to its sheer size.
